# Tubeimoside I Ameliorates Doxorubicin-Induced Cardiotoxicity by Upregulating SIRT3

**DOI:** 10.1155/2023/9966355

**Published:** 2023-01-14

**Authors:** Wei Zhang, Zhixing Fan, Fengyuan Wang, Lin Yin, Jinchun Wu, Dengke Li, Siwei Song, Xi Wang, Yanhong Tang, Congxin Huang

**Affiliations:** ^1^Department of Cardiology, Renmin Hospital of Wuhan University, Wuhan 430060, China; ^2^Cardiovascular Research Institute, Wuhan University, Wuhan 430060, China; ^3^Hubei Key Laboratory of Cardiology, Wuhan 430060, China; ^4^Department of Cardiology, The First College of Clinical Medical Sciences, China Three Gorges University & Yichang Central People's Hospital, Yichang, 443000 Hubei Province, China

## Abstract

Cardiotoxicity linked to doxorubicin (DOX) is primarily caused by inflammation, oxidative stress, and apoptosis. The role of tubeimoside I (TBM) in DOX-induced cardiotoxicity remains ambiguous, despite growing evidence that it could reduce inflammation, oxidative stress, and apoptosis in various diseases. This study was designed to investigate the role of TBM in DOX-induced cardiotoxicity and uncover the underlying mechanisms. H9c2 cell line and C57BL/6 mice were used to construct an in vitro and in vivo model of DOX-induced myocardial injury, respectively. We observed that DOX treatment provoked inflammation, oxidative stress, and cardiomyocyte apoptosis, which were significantly alleviated by TBM administration. Mechanistically, TBM attenuated DOX-induced downregulation of sirtuin 3 (SIRT3), and SIRT3 inhibition abrogated the beneficial effects of TBM both in vitro and in vivo. In conclusion, TBM eased inflammation, oxidative stress, and apoptosis in DOX-induced cardiotoxicity by increasing the expression of SIRT3, suggesting that it holds great promise for treating DOX-induced cardiac injury.

## 1. Introduction

The anthracycline chemotherapeutic drug doxorubicin (DOX) is extensively used to treat neoplastic diseases, including leukemia, lymphoma, and some solid tumors, such as breast, urogenital, endocrine, and stomach cancer [1, 2]. The inhibition of topoisomerase II and DNA intercalation are the main mechanisms by which DOX exerts its antitumor effects [1–3]. Clinical studies have shown that the clinical application of DOX is limited due to its side effects, especially cardiotoxicity [4]. Indeed, there is an increased risk of developing irreversible degenerative cardiomyopathy and ultimately heart failure due to DOX-induced cardiotoxicity [1]. Several mechanisms underlying DOX-induced cardiotoxicity have been studied in depth, including oxidative stress, inflammation, mitochondrial damage, endoplasmic reticulum dysfunction, calcium dyshomeostasis, cardiomyocyte apoptosis, dysregulation of autophagy, and fibrosis [3, 5, 6]. However, there is no specific treatment hitherto for DOX-induced cardiotoxicity. Consequently, the search for an effective treatment remains a top priority.

Sirtuins are nicotinamide adenine dinucleotide (NAD^+^)-dependent class III histone deacetylases, which participate in numerous cellular biological processes, including gene transcription, cell cycle, metabolism, and apoptosis [7–10]. An increasing number of studies indicate that sirtuin-3 (SIRT3) participates in the development of cardiovascular diseases [11–14]. SIRT3 is mainly expressed in highly metabolic organs, such as the heart, brain, kidney, and liver [15–17] and modulates numerous biological processes, including ATP production, catabolism, and reactive oxygen species (ROS) detoxification [18–20]. Furthermore, mounting studies suggested that SIRT3 plays an essential role in regulating cell death, autophagy, and metabolic homeostasis in cardiovascular diseases [21–23]. Growing studies have demonstrated that SIRT3 possesses the ability to attenuate DOX-induced cardiac damage by inhibiting inflammation, oxidative injury, apoptosis, and improving mitochondrial function [24–29].

Tubeimoside I (TBM) is a triterpenoid saponin derived from *tubeimu*, the tuber of *Bolbostemma paniculatum* [30]. Over centuries, *tubeimu* has been used in traditional Chinese medicine to treat acute mastitis, snake bites, detoxication, inflammatory diseases, and tumors. TBM has excellent antitumor properties across cancers in several recent studies [31–33]. Additionally, TBM can antagonize inflammation, improve endothelial cell function, and reduce myocardial ischemia-reperfusion injury by activating SIRT3 [34–36]. However, the effect of TBM on DOX-induced cardiotoxicity remains unclear. Therefore, this study was designed to investigate whether TBM could attenuate DOX-induced cardiac injury and evaluate the role of SIRT3 in this process.

## 2. Materials and Methods

### 2.1. Main Reagents

The following antibodies were used in this study: anti-SIRT3 (#10099-1-AP, purchased from Proteintech Group (Wuhan, China); anti-Nuclear factor E2-related factor 2 (Nrf2, #GTX635826), anti-heme oxygenase 1 (HO-1, #GTX101147), anti-total-NF-*κ*B P65 (T-P65, #GTX102090), antiproliferating cell nuclear antigen (PCNA, #GTX100539), and anti-phospho-NF-*κ*B P65 (P-P65, #GTX133899) were purchased from GeneTex (California, USA); anti-B cell lymphoma 2 (BCL-2, #YT0470), anti-BCL-2-associated X protein (BAX, #YT0455), antitumor necrosis factor *α* (TNF-*α*, #YT4689), and anti-interleukin 6 (IL-6, #YT5348) were purchased from ImmunoWay (Texas, USA); anticleaved caspase3 (C-Caspase3, #9664) was purchased from Cell Signaling Technology (Massachusetts, USA); anti-IL-1*β* (#A1112), anti-NADPH quinone oxidoreductase-1 (NQO1, #A19586), and anti-glyceraldehyde 3-phosphate dehydrogenase (GAPDH, #AC027) were obtained from ABclonal Technology (Wuhan, China). DOX (#HY15142), TBM (#HY-N0890), and 3-(1H-1,2,3-triazol-4-yl) pyridine (3-TYP, #HY108331) were purchased from MedChemExpress (New Jersey, USA); Dihydroethidium (DHE, #D1008) was obtained from Bioscience (Shanghai, China); the TdT-mediated dUTP nick end-labeling (TUNEL) assay kit (#C1088), Annexin V-FITC/PI (propidium iodide) apoptosis detection kit **(**#C1062), Nuclear Protein Extraction Kit (#P0027), and mitochondrial membrane potential assay kit with JC-1 (#C2006) were purchased from Beyotime Biotechnology (Shanghai, China); assay kits for detecting lactate dehydrogenase (LDH, #A02022), creatine kinase isoenzymes (CK-MB, #E00611), cardiac isoform of Troponin T (cTnT, H1494), superoxide dismutase (SOD, #A0011) activity, glutathione peroxidase (GSH-PX, #A005), and malondialdehyde (MDA, #A0031) were purchased from Nanjing Jiancheng Bioengineering Institute (Nanjing, China); the cell counting kit-8 (CCK8) was obtained from TopScience (Shanghai, China). All reagents for western blotting and real-time quantitative PCR tests were purchased from Servicebio Technology (Wuhan, China).

### 2.2. Experimental Animals and Treatments

All of the animal procedures were conducted in accordance with the directives outlined in the Guide for the Care and Use of Laboratory Animals (US National Institutes of Health) and were approved by the Animal Care and Use Committee of Renmin Hospital of Wuhan University (IACUC Issue No. 20220101A). The adult male C57BL/6 mice (8–10 weeks old) were purchased from the Laboratory Animal Center of China Three Gorges University (Yichang, China). These mice had free access to the standard laboratory chow diet and water under a 12 h light-dark cycle in a specific pathogen-free barrier system and were housed at a controlled temperature (20°C–25°C) and humidity (50% ± 5%). After acclimatization to the housing environment for 1 week, mice were randomly divided into the following five groups: control (CON) group, TBM group, DOX group, DOX + TBM group, and DOX + TBM+3-TYP group. Mice in the DOX group, DOX + TBM group, and DOX + TBM+3-TYP group were subjected to a single intraperitoneal injection of DOX (15 mg/kg) to induce acute cardiac injury [37], while the CON group received an equal volume of normal saline. Before DOX injection, mice in the TBM group, DOX + TBM group, and DOX + TBM+3-TYP group were pretreated with TBM (4 mg/kg/2 days) twice [36], and then sequentially received TBM (4 mg/kg/2 days) twice. Meanwhile, DOX + TBM+3-TYP group mice were pretreated with 3-TYP (50 mg/kg/2 days) twice, and then sequentially received TBM (4 mg/kg/2 days) twice. The workflow is illustrated in Figure S1.

### 2.3. Cell Culture and Treatments

H9c2 cells were purchased from Procell Life Technology (Wuhan, China) and were cultured in Dulbecco's modified Eagle's medium supplemented with 10% fetal bovine serum (FBS; Gibco, #1009148) and 1% penicillin/streptomycin, and maintained at 37°C in a humidified incubator with 5% CO_2_. Upon reaching a confluence of 60%–70%, the H9c2 cells were treated with TBM (the optimum concentration determined from CCK8 assay results) or vehicle in the absence or presence of DOX (1 *μ*M) for 24 h. To identify the function of SIRT3, SIRT3 siRNA (si-Sirt3) was transfected into cultured H9c2 cells for 6 h before TBM administration using Lipofectamine RNAiMax (#13778030, Thermo Scientific, USA) according to the manufacturer's instructions. si-Sirt3 was designed using BLOCK-iT™ RNAi Designer (Thermo Scientific) and synthesized by HanBio (Shanghai, China). The target sequence of si-Sirt3 was *CTGAATCGGTACAGAAATC*. Six hours after si-Sirt3 transfection, TBM and DOX were simultaneously added to H9c2 cells for an additional 24 h.

### 2.4. Cell Viability Assay

H9c2 cells were seeded at 2 × 104/ml onto 96-well culture plates for 24 h to achieve a cell density of 60%–70%, before treating with TBM (0, 2, 4, and 8 *μ*M) in the absence or presence of DOX (1 *μ*M) for an additional 24 h. After treatment, 100 *μ*l CCK8 solution was added to each well, and the cells were incubated for 2 h in a 37°C-incubator. Finally, the absorbance of each well was measured at 450 nm with a microplate reader (TECAN, Infinite M200 Pro, Switzerland). The relative cell viability was calculated as the ratio of the absorbance of each treatment group to that of the control group.

### 2.5. Real-Time Quantitative PCR Analysis

Total RNA was extracted with TRIzol reagent and treated with a gDNA Remover to remove genomic DNA; 1 *μ*g of RNA was reversely transcribed into cDNA with the All-in-One First-Strand cDNA Synthesis SuperMix Kit, and quantitative qPCR was performed using 2× Universal Blue SYBR Green Master Mix according to the manufacturer's instructions. The qPCR protocol was performed with the primer sequences listed in Supplementary Table S1 and consisted of 40 cycles of denaturation at 95°C for 15 s, annealing at 60°C for 10 s, and extension at 72°C for 30 s. Measurements were normalized to the mRNA level of endogenous Gapdh. The expression levels of target genes (Il-6, Il-1*β*, Tnf*-α*, Nrf2, Ho-1, Nqo1, Bax, and Bcl-2) were calculated using the 2^–*ΔΔ*Ct^ method. The sequences of target genes are shown in Table S1.

### 2.6. Western Blotting Assay

Total proteins were extracted from H9c2 cells or heart tissues with a protein extraction reagent that contained RIPA lysate, protease-inhibitor, and phosphatase-inhibitor cocktails. Nuclear Protein Extraction Kits were used to extract the nuclear protein in accordance with the manufacturer's instructions. The extracted proteins were then separated on sodium dodecyl sulfate-polyacrylamide gels and transferred to polyvinylidene fluoride membranes. Subsequently, the membranes were blocked with 5% defatted milk for 1 h at room temperature, probed with primary antibody at 4°C overnight. The primary antibodies included rabbit anti-SIRT3 (1 : 1000), rabbit anti-Nrf2 (1 : 1000), rabbit anti-HO-1 (1 : 1000), rabbit anti-NQO1 (1 : 2000), rabbit anti-T-P65 (1 : 1000), rabbit anti-P65 (1 : 1000), rabbit anti-IL6 (1 : 1000), rabbit anti-IL-1*β* (1 : 500), rabbit anti-TNF-*α* (1 : 1000), rabbit anti-BAX (1 : 1000), rabbit anti-BCL-2 (1 : 1000), rabbit anti-C-Caspase3 (1 : 1000), rabbit anti-GAPDH (1 : 3000), and rabbit anti-PCNA (1 : 2000). Thereafter, the membranes were incubated with HRP-conjugated goat anti-rabbit secondary antibodies (1 : 5000) for 1 h at room temperature. Protein bands were visualized by super ECL reagent using a ChemiDoc™ XRS+ system (Bio-Rad Laboratories, Inc., USA) and analyzed using an ImageJ software. The expression level of the target protein was normalized to that of GAPDH or PCNA.

### 2.7. Biochemical Analysis

The levels of LDH, cTnT, and CK-MB in cell medium and mouse serum were examined by an automatic biochemical analyzer (Chemray 800, Rayto). The levels of IL-6, IL-1*β*, TNF-*α*, and MDA, and the activity of SOD and GSH-PX in the cell supernatant and mouse hearts were detected by commercially available kits according to a previous study [37].

### 2.8. Annexin V/PI Test for Cell Apoptosis

H9c2 cells from each treatment group were rinsed thrice with cold PBS, and double-stained with Annexin V-FITC and PI according to the manufacturer's instructions, before incubating in the dark at room temperature for 30 min. Finally, the cells were resuspended in 0.5 ml of the binding buffer and analyzed by flow cytometry (CytoFlex, Beckman Coulter).

### 2.9. Detection of Mitochondrial Membrane Potential by JC-1 Staining

JC-1 dye was used to investigate changes in mitochondrial membrane potential (MMP). According to the manufacturer's instructions, before incubation in the dark at room temperature for 30 min, the treated H9c2 cells were incubated with JC-1 staining working solution in 37°C for 20 min. Finally, a fluorescence microscope (Olympus BX53, Japan) or flow cytometer (CytoFlex, Beckman Coulter) was used to assess the MMP in each group.

### 2.10. DHE Staining for Detecting ROS Generation

Intracellular ROS production was detected by DHE staining in vivo and in vitro. Frozen heart sections were stained with DHE (5 *μ*M), while H9c2 cells were incubated with DHE (5 *μ*M) at 37°C for 30 min in the dark. The images were captured using a fluorescence microscope in vivo, and flow cytometry was used to estimate the ROS level in H9c2 cells.

### 2.11. TUNEL Staining

TUNEL staining was used to determine apoptotic cardiomyocytes in the H9c2 cells and in myocardium according to the manufacturer's instructions. Briefly, cell samples were fixed with 4% paraformaldehyde for 30 min, and then permeabilized with 0.3% Triton X-100 at room temperature for 5 min. For heart samples, 4% paraformaldehyde-fixed myocardial tissues were embedded with paraffin and sectioned into 5 *μ*m thick sections. Sections were mounted onto slides and deparaffinized with xylene. Proteinase K solution without DNase and 0.3% Triton X-100 was used to increase the permeability of heart tissues. Then, TUNEL reaction reagents were used to label the DNA breaks of each specimen in a 37°C-incubator. After rinsing with PBS, the nuclei were labeled with DAPI. Finally, the images were recorded by a fluorescence microscope.

### 2.12. Histological Analysis

After the cardiac function was assessed, mouse hearts were excised, sectioned, and fixed in 10% formalin, or placed in liquid nitrogen for subsequent experiments. Paraffin-embedded sections were deparaffinized in xylene, and rehydrated in a gradient of ethanol to distilled water, before evaluating the histological morphology by hematoxylin and eosin (HE) staining.

### 2.13. Echocardiography

Echocardiographic assessment (Vevo 2100, Visual Sonics Inc., Toronto, Canada) of cardiac functions were performed on mice anesthetized with 1.5% isoflurane. Vevo Analysis software was used to calculate the indices of left ventricular systolic function, including the left ventricular ejection fraction (EF), fractional shortening (FS) values, and heart rate.

### 2.14. Statistical Analysis

All experimental data were analyzed by GraphPad Prism software (version 9.0.2). The results are presented as the mean ± standard deviation (SD). Unpaired two-tailed Student's *t*-test was performed when comparing the two groups, and a one-way analysis of variance (ANOVA) followed by Tukey's post hoc test was performed when comparing multiple groups. *P* values <0.05 were considered statistically significant.

## 3. Results

### 3.1. TBM Attenuated DOX-Induced Injury in H9c2 Cells

**Figure 1:**
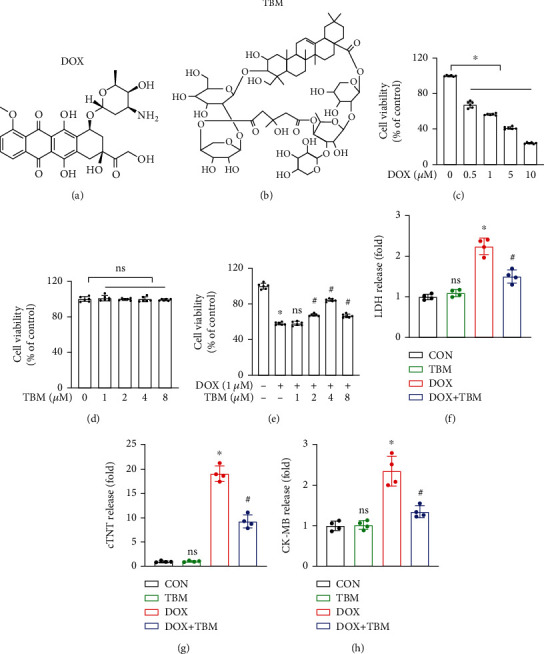
TBM alleviated DOX-induced cellular injury in H9c2 cells. (a, b) The chemical structures of DOX and TBM, respectively. (c, d) Viability of H9c2 cells treated with DOX and TBM for 24 h (*n* = 6). (e) TBM improved the viability of DOX treated H9c2 cells (*n* = 6), ns (not significant) versus the DOX group. (f–h) Levels of LDH, cTNT, and CK-MB in H9c2 cell media (*n* = 4), ns (not significant) versus the CON group. Values indicate the mean ± standard deviation (SD). ∗*P* < 0.05 versus the CON group and the TBM group; ^#^*P* < 0.05 versus the DOX group.

To investigate the effects of TBM on DOX-induced cardiotoxicity, its influence on the viability of H9c2 cells was measured. Figures 1(a) and 1(b) indicate the chemical structures of DOX and TBM, respectively. The viability of H9c2 cells exposed to different concentrations of DOX (0, 0.5, 1, 5, and 10 *μ*M) for 24 h was markedly changed in a concentration-dependent manner (Figure 1(c)), while that of cells administered different concentrations of TBM (0, 1, 2, 4, and 8 *μ*M) for 24 h was not significantly altered (Figure 1(d)), indicating that DOX but not TBM exerted significant cytotoxicity to H9c2 cells under the tested doses. Surprisingly, the viability of H9c2 cells cotreated with TBM (2, 4, and 8 *μ*M) and DOX (1 *μ*M) for 24 h was clearly improved compared to that of H9c2 cells treated only with DOX (1 *μ*M) for 24 h (Figure 1(e)), suggesting that suitable concentrations of TBM could alleviate DOX-induced cytotoxicity. According to the previous studies [38, 39] and our results, 1 *μ*M of DOX and 4 *μ*M of TBM were determined for consequent in vitro studies. In addition, we found that H9c2 cardiomyocytes released LDH, cTNT, and CK-MB to the medium when exposed to DOX, whereas TBM decreased the releases of LDH, cTNT, and CK-MB in the DOX + TBM group (Figures 1(f)–1(h)), indicating that TBM alleviated DOX-induced cytotoxicity in H9c2 cardiomyocytes.

### 3.2. TBM Attenuated Inflammation in H9c2 Cells Treated with DOX

**Figure 2:**
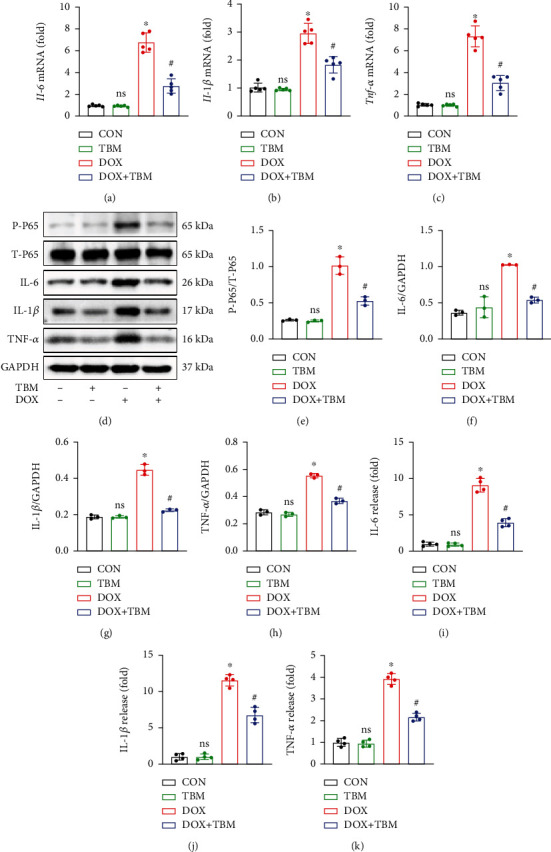
TBM attenuated inflammation in response to DOX treatment in vitro. (a–c) The relative mRNA levels of Il-6, Il-1*β*, and Tnf*-α* in DOX treated H9c2 cells (*n* = 5). (d–h) Western blotting assays of IL-6, IL-1*β*, and TNF-*α* in each group (*n* = 3). (i–k) Levels of IL-6, IL-1*β*, and TNF-*α* in cell supernatant determined by ELISA (*n* = 4). Values indicate the mean ± SD. ∗*P* < 0.05 versus the CON group and the TBM group; ^#^*P* < 0.05 versus the DOX group; ns (not significant) versus the CON group.

As shown in Figures 2(a)–2(c), DOX treatment significantly increased the mRNA levels of Il-6, Il-1*β*, and Tnf*-α* in H9c2 cells, yet TBM treatment inhibited these changes. Western blotting assays indicated that T-P65 showed no obvious difference between the groups, whereas the expression of P-P65 in the DOX group was significantly upregulated (Figures 2(d) and 2(e)). Additionally, the results of both western blotting and ELISA suggested that the protein levels of IL-6, IL-1*β*, and TNF-*α* in the DOX + TBM group were less than those in the DOX group (Figures 2(d) and 2(f–k)). Taken together, the results mentioned above demonstrate that TBM can attenuate inflammation induced by DOX in H9c2 cells.

### 3.3. TBM Efficiently Mitigated DOX-Induced Oxidative Stress In Vitro

**Figure 3:**
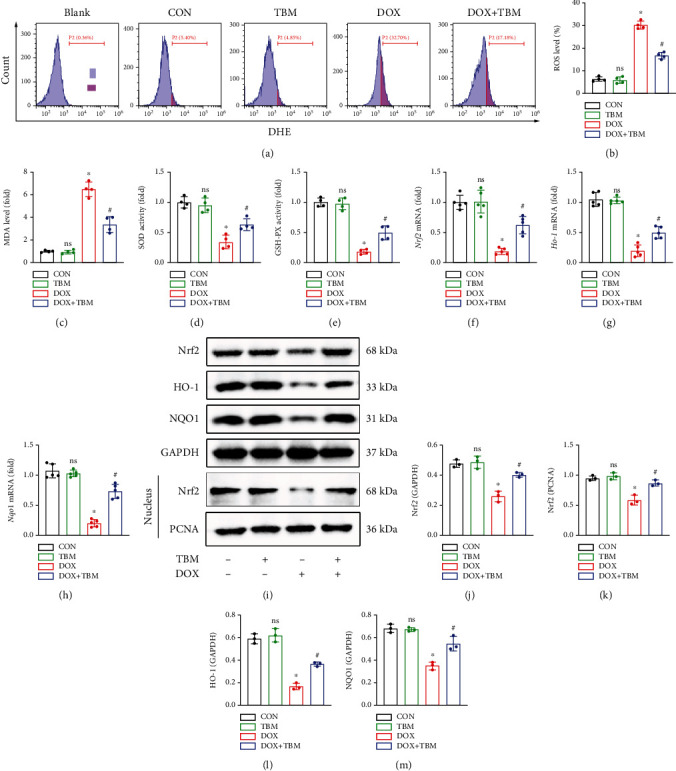
TBM remarkably ameliorated DOX-evoked oxidative stress in H9c2 cells. (a, b) Flow cytometry was used to estimate the ROS levels in H9c2 cells (*n* = 4). (c–e) The level of MDA and the activity of SOD and GSH-PX in H9c2 cells were detected by commercial kits (*n* = 4). (f–h) The mRNA levels of Nrf2, Ho-1, and Nqo1 in H9c2 cells determined by qPCR (*n* = 5). (i–m) Representative western blotting results for Nrf2, HO-1, and NQO1 in vitro (*n* = 3). Values indicate the mean ± SD. ∗*P* < 0.05 versus the CON group and the TBM group; ^#^*P* < 0.05 versus the DOX group; ns (not significant) versus the CON group.

To explore whether TBM can suppress the DOX-induced generation of reactive oxygen species (ROS), the ROS levels were measured in different groups. In a DHE staining determined by flow cytometry, TBM remarkably prevented ROS production in H9c2 cells (Figures 3(a) and 3(b)). Furthermore, the levels of the oxidative stress marker protein MDA were significantly increased in the DOX group, while the activities of the antioxidant enzymes SOD and GSH-PX were significantly decreased in the DOX group, and were clearly reversed by TBM treatment in the DOX + TBM group (Figures 3(c)–3(e)). According to qPCR analysis, the mRNA levels of Nrf2 and its downstream target genes Ho-1, and Nqo1 in the DOX group were significantly less than those in the other groups (Figures 3(f)–3(h)). In line with our qPCR results, the results of western blotting also demonstrated that the protein levels of Nrf2, HO-1, and NQO1 were distinctly reduced in the DOX administration group (Figures 3(i)–3(m)). Additionally, the expression of Nrf2 in nuclei was also reduced by DOX (Figure 3(k)). However, these adverse effects of DOX were prevented by treatment with TBM in the DOX + TBM group (Figures 3(i)–3(m)). Collectively, these results suggested that TBM attenuated the DOX-induced formation of ROS, and oxidative stress damage in vitro.

### 3.4. TBM Protected against DOX-Induced Cardiomyocyte Apoptosis In Vitro

**Figure 4:**
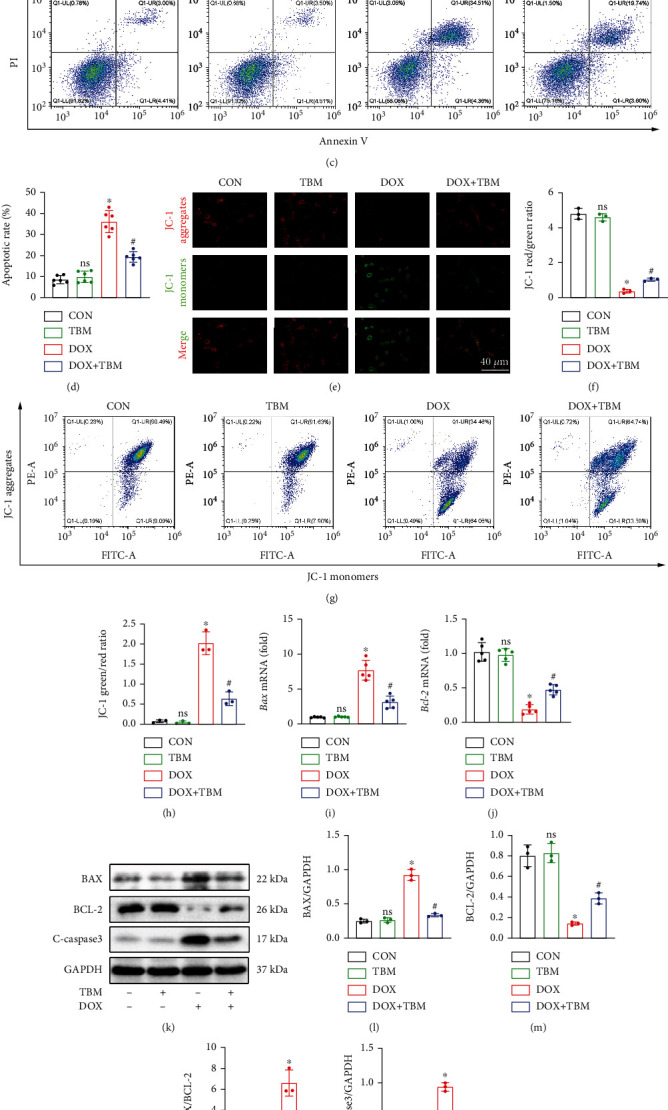
TBM protected H9c2 cells from DOX-induced apoptosis. (a, b) TUNEL staining labeled DNA breaks in H9c2 cells and its statistical analysis (*n* = 3). (c, d) Flow cytometry was also used to evaluate the apoptosis of H9c2 cells (*n* = 6). (e, f) Mitochondrial membrane potential (MMP) was determined by JC-1staining (*n* = 3). (g, h) Flow cytometry was used to estimate MMP in each group (*n* = 3). (i, j) Bax and Bcl-2 mRNA levels tested by qPCR (*n* = 5). (k–o) The expression of BAX, BCL-2, and C-caspase3 at protein level determined by western blotting in vitro (*n* = 3). Values indicate the mean ± SD. ∗*P* < 0.05 versus the CON group and the TBM group; ^#^*P* < 0.05 versus the DOX group; ns (not significant) versus the CON group.

TUNEL staining labels DNA breakpoints with 3′-OH terminals that are well-recognized to detect DNA damage and cell apoptosis. As illustrated in Figures 4(a) and 4(b), TUNEL staining demonstrated that DOX contributed to the production of DNA fragmentation in H9c2 cells, which was remarkably blunted in the presence of TBM. Annexin-V/PI double staining determined by flow cytometry also showed that TBM significantly decreased the apoptotic rate of DOX-treated H9c2 cells (Figures 4(c) and 4(d)). JC-1 staining demonstrated an increase in green fluorescence (JC-1 monomers) and a decrease in red fluorescence (JC-1 aggregates) in the DOX group, indicating adverse changes in MMP, which was reversed by TBM (Figures 4(e)–4(h)). qPCR analysis showed that the mRNA expression level of Bcl-2 and Bax was downregulated and upregulated by DOX, respectively, indicating that DOX administration contributed to obvious apoptosis in vitro, while treatment with TBM ameliorated this pathological change (Figures 4(i) and 4(j)). The suppressive roles of TBM in the apoptosis of DOX-treated H9c2 cells were further verified by western blotting, the results of which revealed that TBM decreased the level of BAX, the BAX/BCL-2 ratio, and cleaved caspase3 (C-Caspase3), and increased the expression of BCL-2 (Figures 4(k)–4(o)).

### 3.5. TBM Alleviated DOX-Induced Cardiotoxicity in Mice

**Figure 5:**
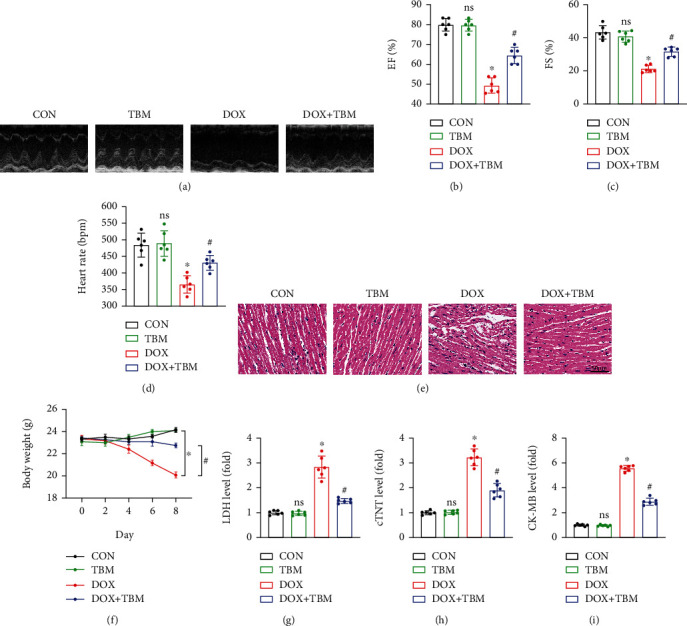
TBM alleviated DOX-induced cardiotoxicity in mice. (a) Representative images of M-mode echocardiography in each group (*n* = 6). (b, c) Statistical analysis of left ventricular ejection fraction (EF) and fractional shortening (FS) (*n* = 6). (d) Heart rate of mice (*n* = 6). (e) HE staining of mouse hearts (*n* = 6). (f) The body weight changes in each group were recorded every other day (*n* = 6). (g–i) The serum levels of LDH, cTNT, and CK-MB in mice (*n* = 6). Values indicate the mean ± SD. ∗*P* < 0.05 versus the CON group and the TBM group; ^#^*P* < 0.05 versus the DOX group; ns (not significant) versus the CON group.

TBM administration alleviated DOX-induced cardiac dysfunction, as demonstrated by the significant increase in the left ventricular ejection fraction (EF), fractional shortening (FS), and heart rate in the DOX + TBM group mice compared to the DOX group (Figures 5(a)–5(d)). Additionally, HE staining showed that TBM could ameliorate the disorganized structure of the myocardium caused by DOX (Figure 5(e)). Previous research has indicated that DOX can decrease the body weight of patients with cancer, which is often indicative of poor prognosis [40]. Unexpectedly, TBM supplementation effectively prevented body weight loss in DOX-injected mice (Figure 5(f)). In addition, DOX injection increased the serum levels of LDH, cTNT, and CK-MB in mice, which were partially reversed by TBM (Figures 5(g)–5(i)). Taken together, it can be concluded that TBM protects the heart from DOX-induced myocardial damage in mice.

### 3.6. TBM Suppressed DOX-Induced Inflammation In Vivo

**Figure 6:**
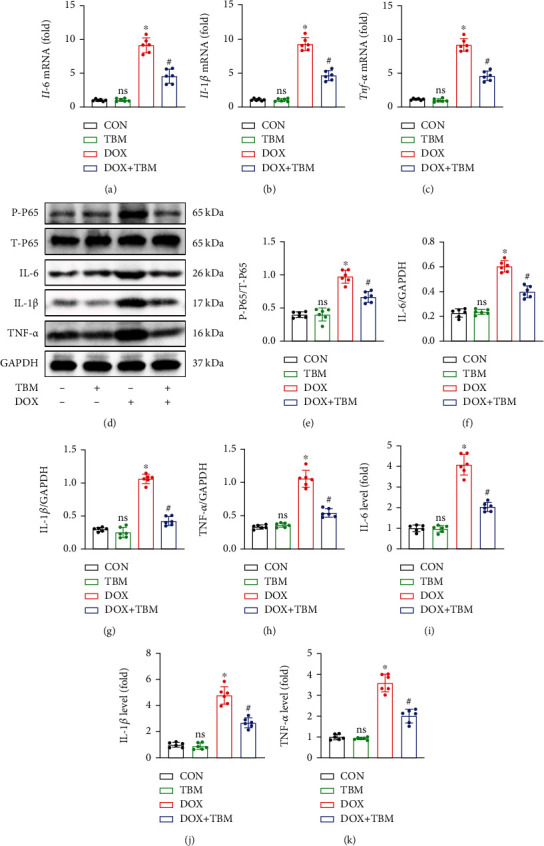
TBM inhibited DOX-induced inflammatory response in vivo. (a–c) The relative mRNA levels of Il-6, Il-1*β*, and Tnf*-α* in mouse hearts (n = 6). (d–h) Western blotting for the detection of NF-*κ*B P65 and inflammatory factors (IL-6, IL-1*β*, and TNF-*α*) in mice (*n* = 6). (i–k) The levels of IL-6, IL-1*β*, and TNF-*α* in mouse hearts determined by ELISA (*n* = 6). Values indicate the mean ± SD. ∗*P* < 0.05 versus the CON group and the TBM group; ^#^*P* < 0.05 versus the DOX group; ns (not significant) versus the CON group.

Given that TBM exerted a protective influence on DOX-induced inflammatory response in vitro, we next conducted an in vivo study to determine whether TBM can protect the heart from DOX-induced inflammation in mice model. As shown in Figures 6(a)–6(c), the results of qPCR indicated that TBM administration remarkably reduced the mRNA expression of Il-6, Il-1*β*, and Tnf*-α*. Furthermore, the P-P65 triggered by DOX was significantly inhibited in the DOX + TBM group (Figures 6(d) and 6(e)). Accordingly, the results of western blotting revealed that TBM suppressed the expression of IL-6, IL-*1β*, and TNF-*α* elicited by DOX treatment (Figures 6(d) and 6(f–h)), which were further consolidated by ELISA assays (Figures 6(i)–6(k)). In short, TBM alleviated inflammation in response to DOX insult in mice.

### 3.7. TBM Ameliorated DOX-Induced Oxidative Stress in Mice

**Figure 7:**
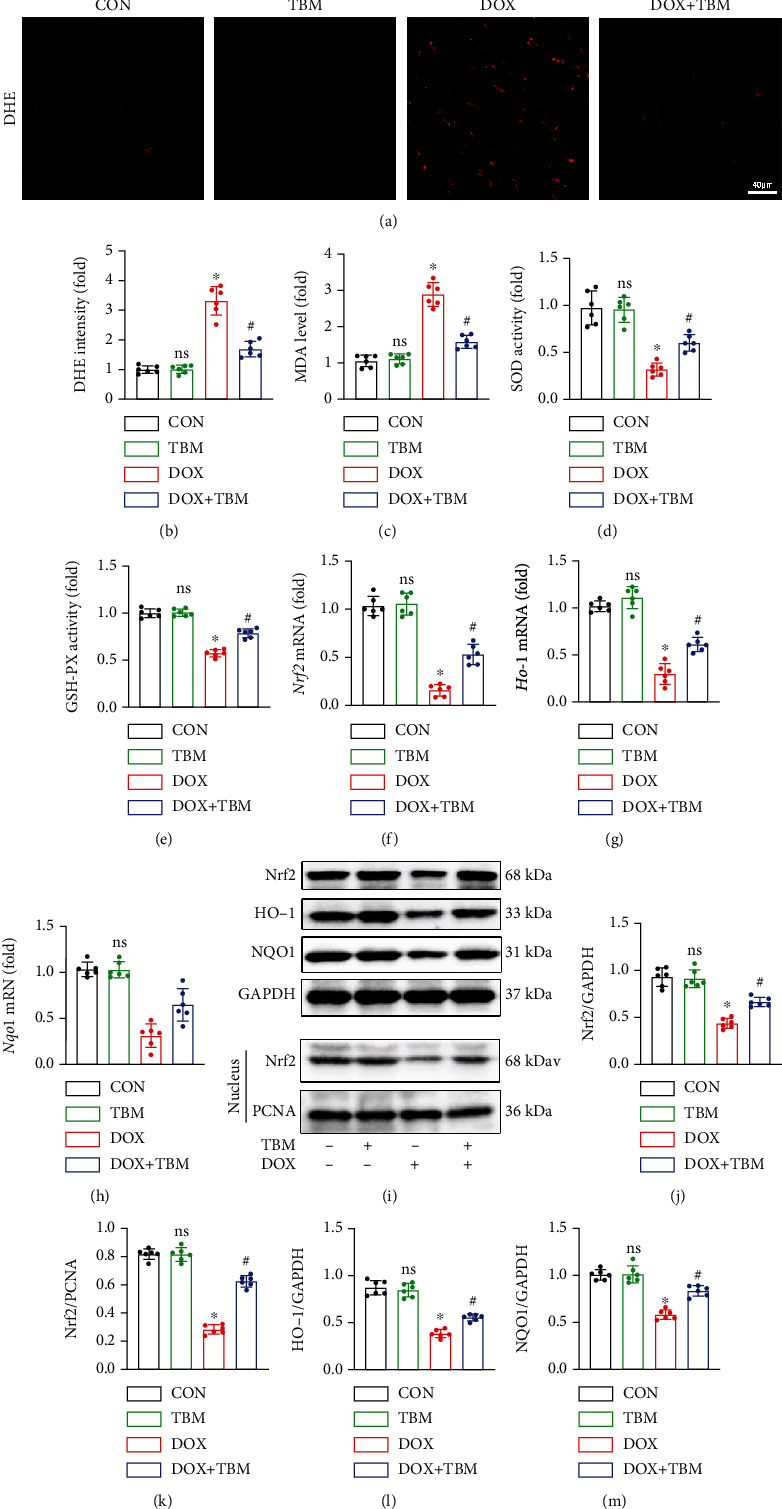
TBM protected against DOX-induced oxidative stress in mice. (a, b) DHE staining indicated the generation of ROS in mice hearts (*n* = 6). (c) The MDA levels in mouse hearts (*n* = 6). (d, e) The activity of SOD and GSH-PX in mouse hearts (*n* = 6). (f–h) The mRNA levels of Nrf2, Ho-1, and Nqo1 in mice determined by qPCR (*n* = 6). (i–m) Western blotting analysis of Nrf2, HO-1, and NQO1 at the protein level (*n* = 6). Values indicate the mean ± SD. ∗*P* < 0.05 versus the CON group and the TBM group; ^#^*P* < 0.05 versus the DOX group; ns (not significant) versus the CON group.

DHE staining demonstrated that DOX distinctly increased the formation of cardiac ROS in mice, which was decreased by TBM administration (Figures 7(a) and 7(b)). We observed the increased levels of MDA in DOX-treated hearts, and decreased activities of SOD and GSH-PX, all of which were inhibited by TBM administration (Figures 7(c)–7(e)). In line with the in vitro results, the mRNA levels of Nrf2 and its downstream target genes (Ho-1 and Nqo1) were notably downregulated in DOX-treated mice, and TBM administration markedly suppressed these effects in response to DOX insult (Figures 7(f)–7(h)). These alterations in protein level were further confirmed by western blotting (Figures 7(i)–7(m)). Taken together, these data illustrated that TBM treatment ameliorated DOX-provoked ROS generation and oxidative damage in mice.

### 3.8. TBM Inhibited DOX-Triggered Cardiomyocyte Apoptosis In Vivo

**Figure 8:**
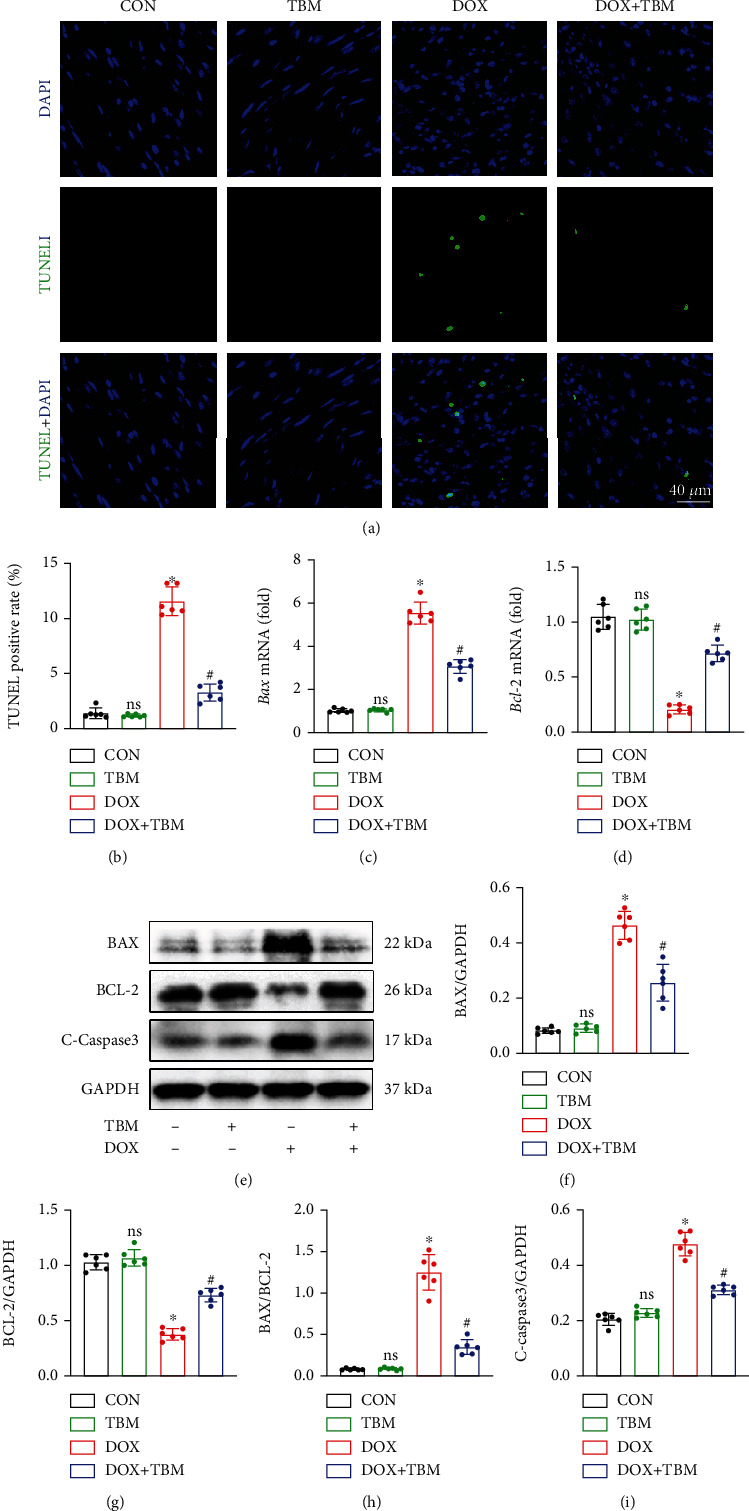
TBM mitigated DOX-provoked cardiomyocyte apoptosis in vivo. (a, b) TUNEL staining represents cardiomyocyte apoptosis in the mouse hearts and its statistical result (*n* = 6). (c, d) The mRNA levels of Bax and Bcl-2 determined by qPCR analysis (*n* = 6). (e–i) Western blotting assays for the detection of BAX, BCL-2, and C-Caspase3 expression (*n* = 6). Values indicate the mean ± SD. ∗*P* < 0.05 versus the CON group and the TBM group; ^#^*P* < 0.05 versus the DOX group; ns (not significant) versus the CON group.

We next conducted an in vivo experiment in mice to confirm the antiapoptotic effects of TBM on DOX-induced myocardial damage. In agreement with in the vitro study, TUNEL staining suggested that TBM decreased the cardiomyocyte apoptosis in the DOX + TBM group mice compared to that in the DOX group (Figures 8(a) and 8(b)). Simultaneously, qPCR analysis showed that the mRNA expression of Bax and Bcl-2 was notably increased and decreased, respectively, in DOX-injected mice. Both changes were remarkably blunted in the DOX + TBM group (Figures 8(c) and 8(d)). Western blotting was also performed to verify the antiapoptotic role of TBM. As expected, TBM significantly inhibited the expression of BAX and C-Caspase3, and distinctly promoted the expression of the BCL-2, reducing the BAX/BCL-2 ratio in DOX-treated mice (Figures 8(e)–8(i)). Collectively, these results indicated that TBM could ameliorate the myocardial injury caused by DOX in mice.

### 3.9. TBM Prevented DOX-Evoked Cellular Damage by Upregulating SIRT3 In Vitro

**Figure 9:**
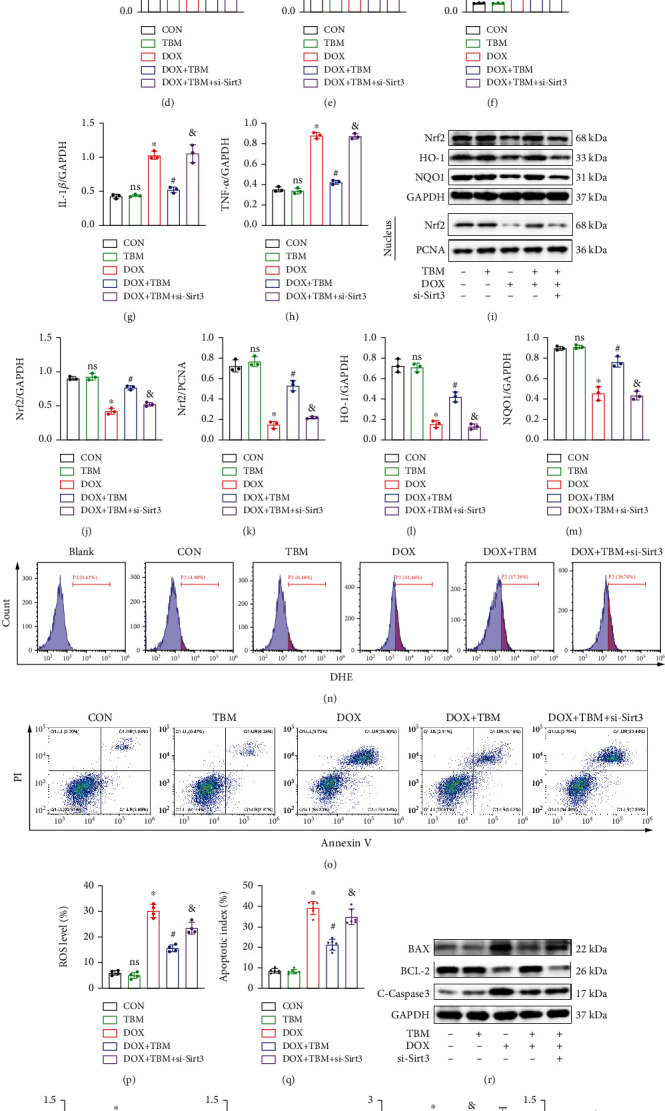
TBM prevented DOX-evoked cellular damage by upregulating SIRT3 in H9c2 cells. (a, b) si-SIRT3 inhibited the expression of SIRT3 as determined by western blotting (n = 4). (c–h) The expression of SIRT3, P-P65, T-P65, IL-6, IL-1*β*, and TNF-*α* was estimated by western blotting in H9c2 cells (*n* = 3). (i–m) Western blotting was used to detect the expression of Nrf2, HO-1, and NQO1 in vitro (*n* = 3). (n, p) si-Sirt3 partially reversed the inhibitory roles of TBM in DOX-induced ROS production (*n* = 4). (o, q) Flow cytometry analysis demonstrated that SIRT3 inhibition blunted the beneficial effects of TBM on DOX-evoked apoptosis (*n* = 6). (r–v) Western blotting was used to test the expression of apoptosis related proteins (BAX, BCL-2, and C-Caspase3) in H9c2 cells after si-Sirt3 treatment (*n* = 3). Values indicate the mean ± SD. ∗*P* < 0.05 versus the CON group and the TBM group; ^#^*P* < 0.05 versus the DOX group; ns (not significant) versus the CON group; ^&^*P* < 0.05 versus the DOX + TBM + siSIRT3 group.

To investigate whether the protective effect of TBM on DOX-induced cardiotoxicity was achieved through modulating SIRT3 expression, H9c2 cells were transfected with si-Sirt3 in the presence of DOX and TBM, and the efficiency of si-Sirt3 was confirmed by western blotting (Figures 9(a) and 9(b)). We also observed that the expression of SIRT3 in the DOX + TBM group was significantly higher than that in the DOX group and DOX + TBM + si-Sirt3 group, indicating that TBM promoted the expression of SIRT3, which could be suppressed by si-Sirt3 (Figures 9(c) and 9(d)). As illustrated in Figures 9(c) and 9(e), incubation with TBM notably attenuated DOX-elicited phosphorylation of T-P65, which was eliminated by si-Sirt3. Synchronously, the suppressive effect of TBM on the production of proinflammatory cytokines (IL6, IL-1*β*, and TNF-*α*) was also blunted by si-Sirt3 (Figures 9(c) and 9(f–h)). Furthermore, the results of western blotting showed that si-Sirt3 distinctly inhibited the beneficial roles of TBM on DOX-induced reduction of Nrf2, HO-1, and NQO1 (Figures 9(i)–9(m)). The increased generation of ROS triggered by DOX was also mitigated by TBM, but these protective roles were partially abolished after si-Sirt3 transfection (Figures 9(n) and 9(p)). Furthermore, JC-1 staining indicated that si-Sirt3 decreased the MMP-protective effect provided by TBM in DOX-treated H9c2 cells (Figure S2A). Our further study showed that si-Sirt3 notably reversed the inhibitory effect of TBM on DOX-induced apoptosis in H9c2 cells (Figures 9(o) and 9(q), and Figure S2B). The changes in the expression of the antiapoptotic protein BCL-2 and proapoptotic proteins (BAX and C-Caspase3) also confirmed that si-Sirt3 blocked the protective roles of TBM in DOX-induced cellular injury (Figures 9(r)–9(v)). Collectively, these results illustrated that TBM protected against DOX-induced cellular damage by upregulating SIRT3 in H9c2 cells.

### 3.10. SIRT3 Inhibition Alleviated the Beneficial Effects of TBM on DOX-Induced Cardiac Injury in Mice

**Figure 10:**
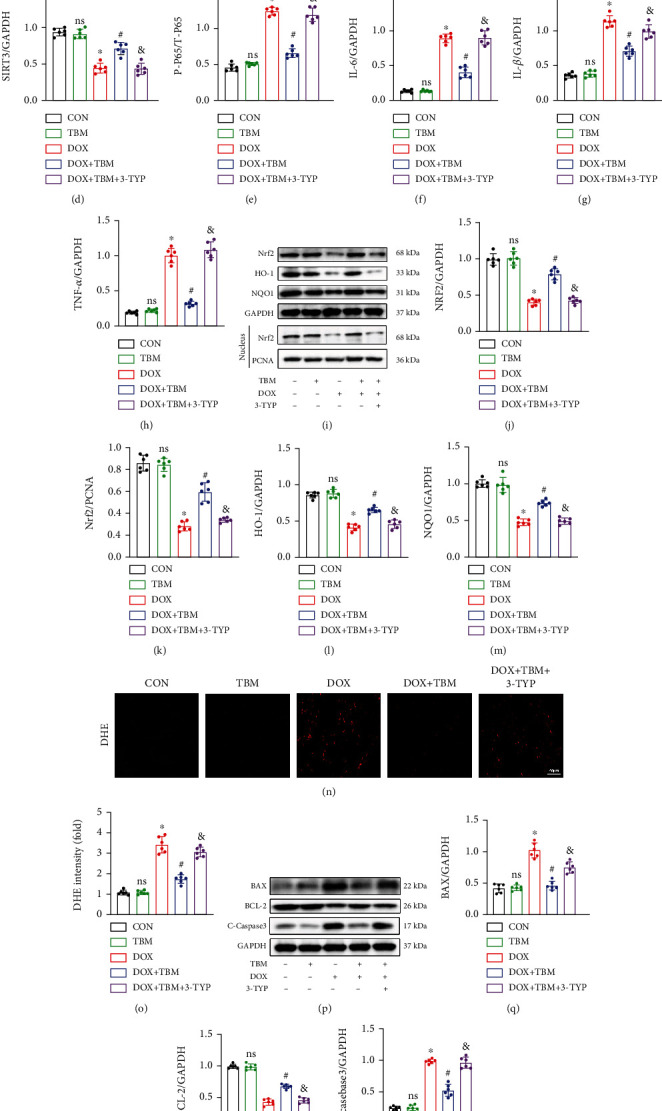
SIRT3 inhibition alleviated the beneficial effects of TBM on DOX-induced cardiac injury in mice. (a, b) 3-TYP inhibited the expression of SIRT3 in mouse hearts (*n* = 6). (c–h) The expression of SIRT3, P-P65, T-P65, IL-6, IL-1*β*, and TNF-*α* evaluated by western blotting in mice (*n* = 6). (i–m) Western blotting was used to examine the expression of Nrf2, HO-1, and NQO1 in mouse hearts (*n* = 6). (n, o) DHE staining illustrated the generation of ROS after SIRT3 inhibition in mouse hearts (*n* = 6). (p–s) Western blotting was used to detect the expression of the antiapoptotic protein BCL-2 and proapoptotic proteins (BAX and C-caspase3) in vivo (*n* = 6). Values indicate the mean ± SD. ∗*P* < 0.05 versus the CON group and the TBM group; ^#^*P* < 0.05 versus the DOX group; ns (not significant) versus the CON group; ^&^*P* < 0.05 versus the DOX + TBM+3-TYP group.

The mice were then treated with 3-TYP (a selective inhibitor of SIRT3) according to a previous study [36] to further evaluate the involvement of SIRT3 in mice injected with DOX. 3-TYP suppressed the expression of SIRT3 protein as indicated in Figures 10(a)–10(d). TBM treatment significantly restrained the phosphorylation of T-P65 and reduced the levels of myocardial IL6, IL-1*β*, and TNF-*α* in the hearts of DOX-injected mice, but had no significant impact on the 3-TYP-treated mice (Figures 10(c) and 10(e–h). TBM-mediated antioxidant effects were markedly blocked after SIRT3 inhibition, as verified by the results of western blotting and DHE staining in mouse hearts (Figures 10(i)–10(o)). Simultaneously, the increase of BCL-2 and decrease in BAX and C-Caspase3 regulated by TBM were abrogated in the presence of 3-TYP, as determined by western blotting (Figures 10(p)–10(s)). Taken together, these findings confirmed that SIRT3 suppression blunted the cardioprotective roles of TBM in vivo; in other words, TBM ameliorated doxorubicin-induced cardiotoxicity by elevating the expression of SIRT3.

## 4. Discussion

It is well-known that DOX is a potent medication against tumor progression in the clinic, and significantly improves the survival rate of patients with cancer; however, many patients suffer from DOX-induced cardiac injury with left ventricular dysfunction and even heart failure, which severely restricts its clinical application [2, 41]. Multiple pathological processes, including the inflammatory response, oxidative stress, autophagy, apoptosis, pyroptosis, and mitochondrial injury, have been reported to be involved in the myocardial damage caused by DOX [2, 3, 42, 43]. Increasing studies have suggested that targeted therapy for these pathological processes is beneficial in mitigating DOX-induced cardiotoxicity [3].

Recently, the triterpenoid saponin TBM, derived from *tuberimu*, a tuber of *Bolbostemma paniculatum*, has attracted much attention because of its multiple pharmacological effects, including antitumor activities [31], anti-inflammatory action [44], the ability to promote angiogenesis [45], and to improve endothelial function [46]. In addition, TBM plays critical protective roles in various cardiovascular diseases [36, 46]. TBM administration protects against myocardial ischemia-reperfusion injury by decreasing oxidative stress [36]. Cheng et al. [46] reported that TBM protected against sepsis-induced cardiac dysfunction. However, it still remains unclear whether TBM exerts a cardioprotective effect on DOX-induced cardiotoxicity. As expected, we found that TBM treatment significantly inhibited inflammation, oxidative stress, and apoptosis in DOX-treated mice and H9c2 cells.

Numerous studies have demonstrated that excessive inflammation is one of the main features of DOX-induced acute cardiotoxicity [37, 47, 48]. Inflammatory cells are stimulated and proinflammatory cytokines are released following the administration of DOX, thereby amplifying the inflammatory cascade [48–50]. In response to extracellular stimulation, NF-*κ*B P65 triggers the formation of inflammatory cytokines [51]. Multiple proinflammatory factors, including IL-6, IL-1*β*, and TNF-*α*, participate in the pathological process of DOX-elicited heart damage [37, 52–54]. We next investigated the role of TBM in DOX-induced inflammation. Our result showed that DOX significantly elevated the phosphorylation level of P65 and the expression of IL-6, IL-1*β*, and TNF-*α*. Treatment with TBM not only suppressed the level of P-P65 but also decreased DOX-induced upregulation of IL-6, IL-1*β*, and TNF-*α*. Additionally, TBM administration reduced the mRNA levels of these proinflammatory factors in mouse hearts and H9c2 cells exposed to DOX.

DOX-evoked cardiotoxicity is also characterized by oxidative stress-related injury [42]. The heart expresses low levels of antioxidant enzymes, rendering it particularly vulnerable to oxidative damage [55]. It has been reported that TBM administration protects against myocardial ischemia-reperfusion injury by inhibiting oxidative stress [36]. Thus, we further explored whether TBM could relieve oxidative injury following DOX treatment. Our results demonstrated that TBM administration decreased the levels of ROS and MDA, but increased the activities of SOD and GSH-PX in DOX-treated mice and H9c2 cells. Nrf2 translocates into the nucleus to initiate the transcription of multiple antioxidant genes (Ho-1 and Nqo1) [56]. In the present study, cardiac Nrf2 transcription and expression levels were decreased upon DOX treatment, which was prevented by TBM administration. TBM also elevated the expression of Nrf2 in the nucleus and significantly increased the mRNA levels of Ho-1 and Nqo1 and their protein expression. These results illustrate that TBM could antagonize oxidative injury triggered by DOX, both in vitro and in vivo.

Cardiomyocyte apoptosis is another critical pathogenic mechanism in DOX-induced cardiomyopathy [57]. Many scholars believe that apoptosis is the major cause of cardiac dysfunction in DOX-induced myocardial damage [3, 43, 57]. We found that TBM improved cardiac dysfunction in mice after DOX insult. Consistent with previous literature [58–60], DOX triggered apoptosis of H9c2 cells and of cardiomyocytes in mouse hearts. However, the administration of TBM mitigated DOX-triggered cardiomyocyte apoptosis, as evidenced by the decreased apoptotic index, elevated antiapoptotic protein (BCL-2), and reduced proapoptotic proteins (BAX and C-Caspase3) in vitro and in vivo. The permeability of the mitochondrial membrane is altered during apoptosis, which leads to a reduction in MMP [61]. TBM incubation significantly restored the impaired MMP in DOX-treated H9c2 cells, indirectly demonstrating that TBM alleviated DOX-induced cardiomyocyte apoptosis.

SIRT3 is a class III histone deacetylases situated in the mitochondria and is involved in multiple biological functions [62]. It has been reported that SIRT3 participates in the remission of various diseases, including myocardial infarction, atherosclerosis, neuron ischemia, hypertrophy, and diabetic cardiomyopathy [11]. Additionally, growing studies have identified that SIRT3 plays a critical protective role in DOX-induced cardiomyopathy. Previous research conducted by Pillai et al. [25] demonstrated that SIRT3 protects against mitochondrial DNA damage and blocked the development of doxorubicin-induced cardiomyopathy in mice. Cheung et al. [24] reported that SIRT3 attenuates DOX-induced oxidative stress and improves mitochondrial respiration in H9c2 cardiomyocytes. Coelho et al. [26] reported that SIRT3 modulation mediated by berberine protects against DOX-induced injury in H9c2 cardiomyoblasts. Liu et al. [28] found that LCZ696 protects against doxorubicin-induced cardiotoxicity by inhibiting ferroptosis via activation of the AKT/SIRT3/SOD2 signaling pathway. Recently, Liu and Zhao [27] confirmed that spinacetin alleviates DOX-induced cardiotoxicity by initiating protective autophagy through SIRT3/AMPK/mTOR pathways. Moreover, SIRT3 prevents DOX-induced dilated cardiomyopathy by modulating protein acetylation and oxidative stress [29]. These studies suggested that SIRT3 plays an important protective role in DOX-induced myocardial injury, indicating that SIRT3 is a potential target for treating DOX-induced cardiomyopathy.

SIRT3 inhibition promotes the expression of P-P65 and upregulates target genes of proinflammatory cytokines [63]. SIRT3 is also involved in scavenging ROS and inhibits oxidative stress in cardiovascular diseases [14]. To determine whether TBM exerts a cardioprotective effect on DOX-induced cardiac damage by modulating SIRT3 expression, si-Sirt3, and 3-TYP were used in vitro and in vivo, respectively, to suppress SIRT3 expression. We observed that TBM markedly restrained the expression of P-P65 and proinflammatory factors (IL-6, IL-1*β*, and TNF-*α*) by increasing the expression of SIRT3. Our data demonstrated that TBM significantly suppressed the generation of ROS and remarkably restored the level of the antioxidative system (Nrf2, HO-1, NQO1, SOD, and GSH-PX) in DOX-treated models, while SIRT3 inhibition partially reversed the antioxidative protection conferred by TBM in DOX-treated mice and H9c2 cells. Additionally, TBM exerted antiapoptotic effects on DOX-elicited cardiac injury, which might be achieved by combining its anti-inflammatory and antioxidative stress effects, and finally played a cardioprotective role in DOX-induced cardiotoxicity. Therefore, the antiapoptotic effect of TBM was also reversed after SIRT3 inhibition.

In this study, we illustrated that TBM alleviated inflammation, oxidative stress, and apoptosis in DOX-induced cardiotoxicity by upregulating SIRT3. Nevertheless, the present study still had several limitations. First, H9c2 cardiomyocytes were used to construct an in vitro model of DOX-induced myocardial injury according to previous studies [38, 39, 64]. Although the H9c2 cell line has been employed in many cardiovascular disease models, there are some shortcomings, including its capability of cell division, loss of specific cardiac features of adult cardiomyocytes, and resistance to toxic injury, all of which may have a significant impact on extrapolating the in vitro results to in vivo mechanisms [65]. Therefore, primary cardiomyocytes should be employed in future in vitro studies. Second, SIRT3 ameliorates DOX-induced myocardial injury by multiple mechanisms, however, we only illustrated that TBM alleviates DOX-induced cardiotoxicity through increasing SIRT3 expression; thus, the precise mechanism underlying TBM-mediated SIRT3 expression needs to be further investigated.

## 5. Conclusion

TBM inhibited inflammation, oxidative stress, and apoptosis in DOX-induced cardiotoxicity by upregulating SIRT3. Our findings suggested that TBM represented a potential therapeutic medication for treating DOX-induced cardiac injury.

## Data Availability

All authors confirmed that the data supporting the findings of the study were provided within the manuscript and the supplementary files.

## Supplementary Materials

Supplementary Materials Table S1: the primer sequences used in this present study. Figure S1: the workflow of animal experiments in this study. Figure S2A: JC-1 staining indicated that TBM improved mitochondrial membrane potential (MMP) in DOX-treated cells, which was partially abolished by si-SIRT3. Figure S2B: TUNEL staining demonstrated that si-SIRT3 partially reversed the protective role of TBM in DOX-treated H9c2 cells.
