# Supplementary material for: Tubeimoside I Ameliorates Doxorubicin-Induced Cardiotoxicity by Upregulating SIRT3
Source: Oxid Med Cell Longev. 2023 Jan 14;2023:9966355. doi: 10.1155/2023/9966355 (PMC9867588; doi:10.1155/2023/9966355)
Supplement: Supplementary Materials — Table S1: the primer sequences used in this present study. Figure S1: the workflow of animal experiments in this study. Figure S2A: JC-1 staining indicated that TBM improved mitochondrial membrane potential (MMP) in DOX-treated cells, which was partially abolished by si-SIRT3. Figure S2B: TUNEL staining demonstrated that si-SIRT3 partially reversed the protective role of TBM in DOX-treated H9c2 cells. [file 9966355.f1.docx]

**Table S1**. The primer sequences used in this present study

| **Rat** | **Gene** | **Primer sequences (5'to3')** |
| --- | --- | --- |
|  | *Il-6* | Forward: CCTACCCCAACTTCCAATGCT |
|  |  | Reverse: GGTCTTGGTCCTTAGCCACT |
|  | *Il-1β* | Forward: TTGAGTCTGCACAGTTCCCC |
|  |  | Reverse: TCCTGGGGAAGGCATTAGGA |
|  | *Tnf-α* | Forward: CACACTGCCGCTTCCTCTAT |
|  |  | Reverse: CTGAGGGATGCCTGTTACCG |
|  | *Nrf2* | Forward: GCCTTCCTCTGCTGCCATTAGTC |
|  |  | Reverse: TGCCTTCAGTGTGCTTCTGGTTG |
|  | *Ho-1* | Forward: TGCACATCCGTGCAGAGAAT |
|  |  | Reverse: CTGGGTTCTGCTTGTTTCGC |
|  | *Nqo1* | Forward: AGGATGGGAGGTGGTCGAATCTG |
|  |  | Reverse: GCCTTCCTTATACGCCAGAGATGAC |
|  | *Bax* | Forward: AGACACCTGAGCTGACCTTGGAG |
|  |  | Reverse: TTCATCGCCAATTCGCCTGAGAC |
|  | *Bcl-2* | Forward: TGGAGAGCGTCAACAGGGAGATG |
|  |  | Reverse: GTGCAGATGCCGGTTCAGGTAC |
|  | *Gapdh* | Forward: GACATGCCGCCTGGAGAA AC |
|  |  | Reverse: AGCCCAGGATGCCCTTTAGT |
| **Mouse** | **Gene** | **Primer sequences (5'to3')** |
|  | *Il-6* | Forward: TCCAGTTGCCTTCTTGGGAC |
|  |  | Reverse: GTGTAATTAAGCCTCCGACTTG |
|  | *Il-1β* | Forward: TGCCACCTTTTGACAGTGATG |
|  |  | Reverse: TGTGCTGCTGCGAGATTTGA |
|  | *Tnf-α* | Forward: AGGCACTCCCCCAAAAGATG |
|  |  | Reverse: CCACTTGGTGGTTTGTGAGTG |
|  | *Nrf2* | Forward: ATGGACTTGGAGTTGCCACC |
|  |  | Reverse: CACACACTTTCTGCGTGCTC |
|  | *Ho-1* | Forward: ACAGCCCCACCAAGTTCAAA |
|  |  | Reverse: GCCAGGCAAGATTCTCCCTT |
|  | *Nqo1* | Forward: CATTGCAGTGGTTTGGGGTG |
|  |  | Reverse: TCTGGAAAGGACCGTTGTCG |
|  | *Bax* | Forward: TGCAGAGGATGATTGCTGAC |
|  |  | Reverse: GATCAGCTCGGGCACTTTAG |
|  | *Bcl-2* | Forward: GGTGGTGGAGGAACTCTTCA |
|  |  | Reverse: ATGCCGGTTCAGGTACTCAG |
|  | *Gapdh* | Forward: AGGTCGGTGTGAACGGATTTG |
|  |  | Reverse: TGTAGACCATGTAGTTGAGGTCA |


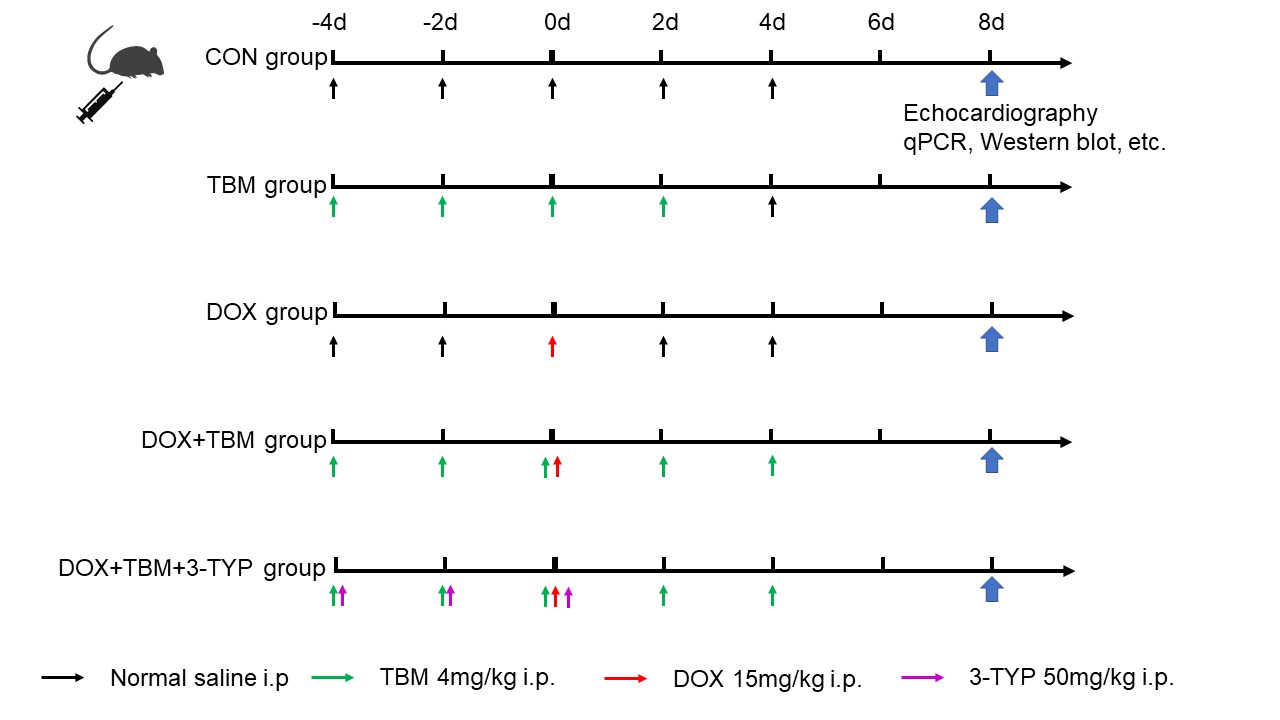


**Figure S1**. The workflow of animal experiments in this study.


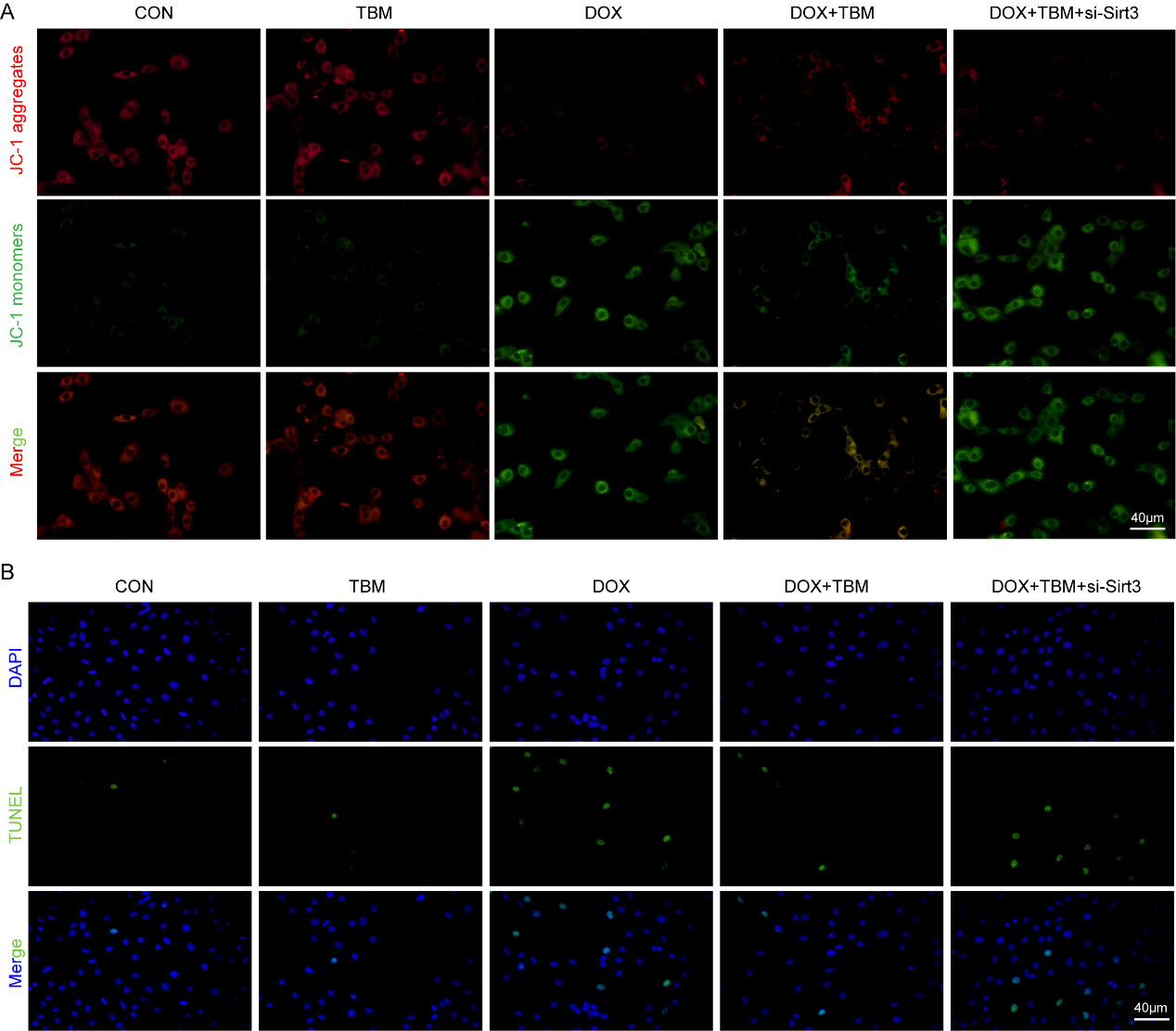


**Figure S2**. SIRT3 inhibition alleviated the beneficial effects of TBM on DOX-induced injury in H9c2 cells. **A** JC-1 staining indicated that TBM improved mitochondrial membrane potential (MMP) in DOX-treated cells, which was partially abolished by si-Sirt3. **B** TUNEL staining demonstrated that si-Sirt3 partially reversed the protective role of TBM in DOX-treated H9c2 cells.
